# A Case Report on Multiple Impacted Teeth: A Three-Dimensional Radiological Evaluation by Cone-Beam Computed Tomography

**DOI:** 10.7759/cureus.116292

**Published:** 2026-09-15

**Authors:** Karthik Rajaram Mohan, Ramachandra Reddy Gowda Venkatesha, Saramma Mathew Fenn

**Affiliations:** 1 Oral Medicine and Radiology, Vinayaka Mission's Sankarachariyar Dental College, Vinayaka Mission's Research Foundation (Deemed to be University), Salem, IND

**Keywords:** cone-beam computed tomography (cbct), dilaceration, impacted teeth, odontogenic cysts, pericoronitis

## Abstract

Multiple impacted teeth refer to a clinical condition where more than one tooth fails to erupt beyond its chronological age of eruption and remains partially or fully submerged in the jawbone. Multiple impacted teeth commonly arise from conditions such as cleft palate and cleft lip, genetically linked syndromes, and pathological conditions like mucopolysaccharidosis. Impacted teeth, or wisdom teeth, commonly occur in the maxillary or mandibular third molars. Still, other teeth, such as maxillary canine and mandibular premolars, can also be affected. Our case discusses an incidental finding of multiple impacted teeth: 15 impacted teeth in the maxilla and 12 impacted teeth in the mandible in a 23-year-old male radiologically evaluated by CBCT.

## Introduction

Multiple impacted teeth occur from varied etiological factors, such as genetic abnormality-related syndromes, endocrine disturbances, such as hypothyroidism and hypoparathyroidism, and metabolic disorders like mucopolysaccharidoses [1,2]. The frequency of multiple impacted teeth occurs in the region of the permanent mandibular third molar followed by the maxillary third molar accompanied by maxillary canine and maxillary second premolars [3]. The frequency of mandibular to maxillary third molar among permanent dentition ranges between 16.7% and 73.82% [3]. Multiple impacted teeth can lead to root resorption, deflection of erupting adjacent teeth, and prone to the formation of cysts such as dentigerous cysts [3]. The incidence of impacted supernumerary teeth ranges from 0.1-1% among the general population of Brooklyn, New York, USA [4]. The treatment of such impacted teeth is determined based on clinical symptoms, tooth location, and an in-depth examination of the impact of these impacted teeth on adjacent nearby teeth [4]. About 18.4% of impacted supernumerary teeth occur in the age group of 21-30 in South India [5]. Various research studies reported the prevalence of impacted teeth as 6.67%, 1-3%, 24.3%, and 1.84% among the Guangzhou Chinese, Caucasian, Jazan, and Spanish populations [6-9].

## Case presentation

A 29-year-old male reported a complaint of an unesthetic appearance due to the absence of teeth. The medical history was non-contributory. His family history revealed none of his parents had similar multiple impacted supernumerary teeth. His ophthalmological and neurological examination did not reveal any significant changes. His chest X-ray revealed no abnormalities such as vertebral anomalies, scoliosis, and the absence of a clavicle. The provisional diagnosis was made as anodontia. The differential diagnosis includes multiple impacted teeth without any associated syndromes or underlying endocrine disorders such as hypothyroidism and hypoparathyroidism. His biochemical investigations revealed that serum PTH was 49 pg/mL (normal range 10-55 pg/mL), serum thyroid-stimulating hormone (TSH) was 0.6 mU/L (normal range = 0.4-4 mU/L), and serum calcium was 9.8 mg/dl (normal range = 9-11 mg/dl), which were within biological ranges. The patient was subjected to cone-beam computed tomography (CBCT) due to its ability to image impacted teeth and their location in three-dimensional planes, compared to conventional orthopantomography, which provides information only in two dimensions. Radiological evaluation by the Carestream 9600s CBCT machine with exposure parameters of 90 kV, 6 mA, and 12.116 msecs revealed 15 multiple impacted teeth in the maxilla with dilaceration of right maxillary second molar #2 and impacted left mandibular premolars #20, 21 (universal numbering system) and 12 impacted teeth in the mandible (Figures 1A, 1B).

**Figure 1:**
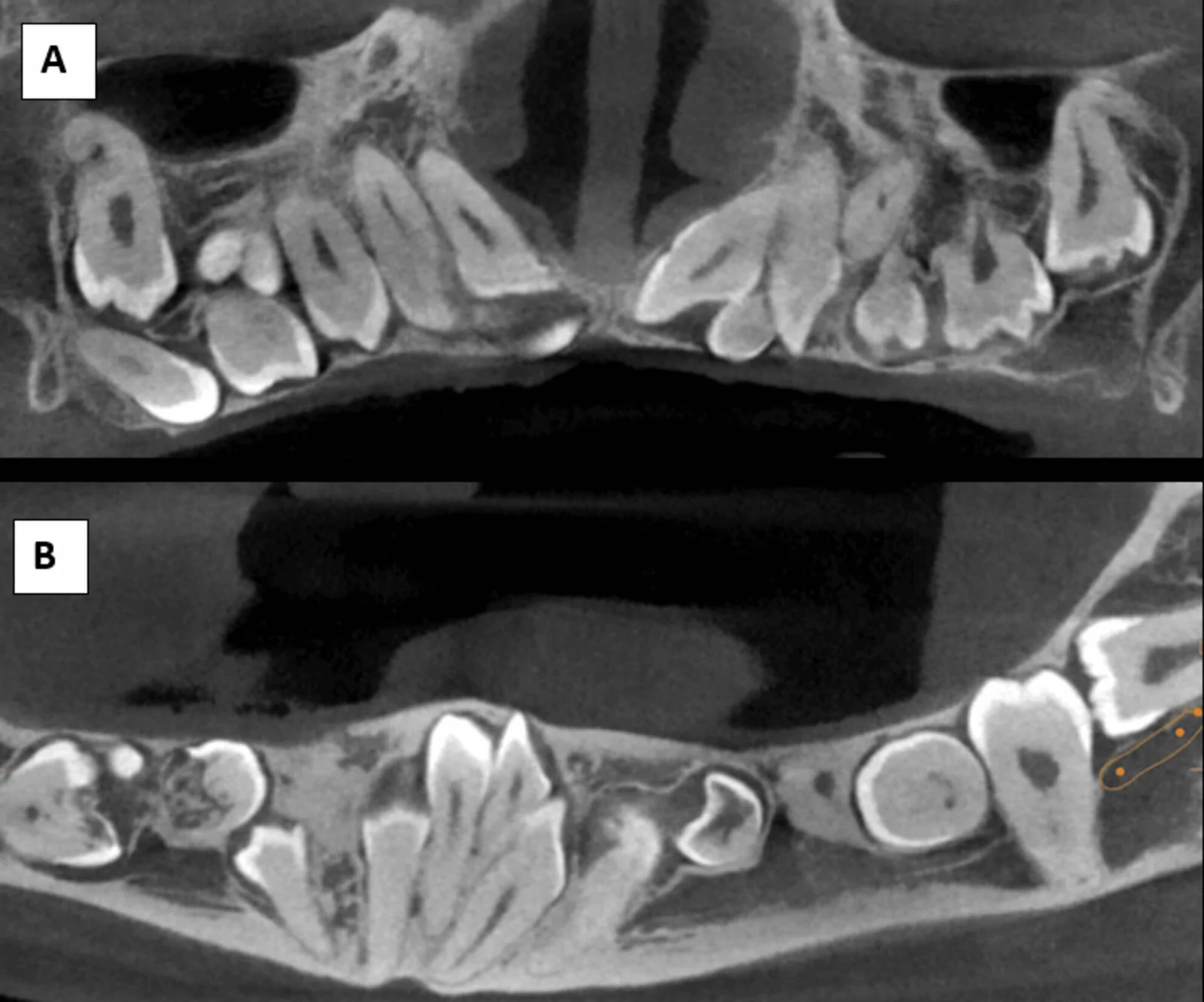
CBCT - panoramic section images; A) Maxilla and B) Mandible revealed multiple impacted teeth CBCT: cone-beam computed tomography

CBCT axial section revealed multiple impacted teeth in the maxilla and mandible (Figures 2A, 2B).

**Figure 2:**
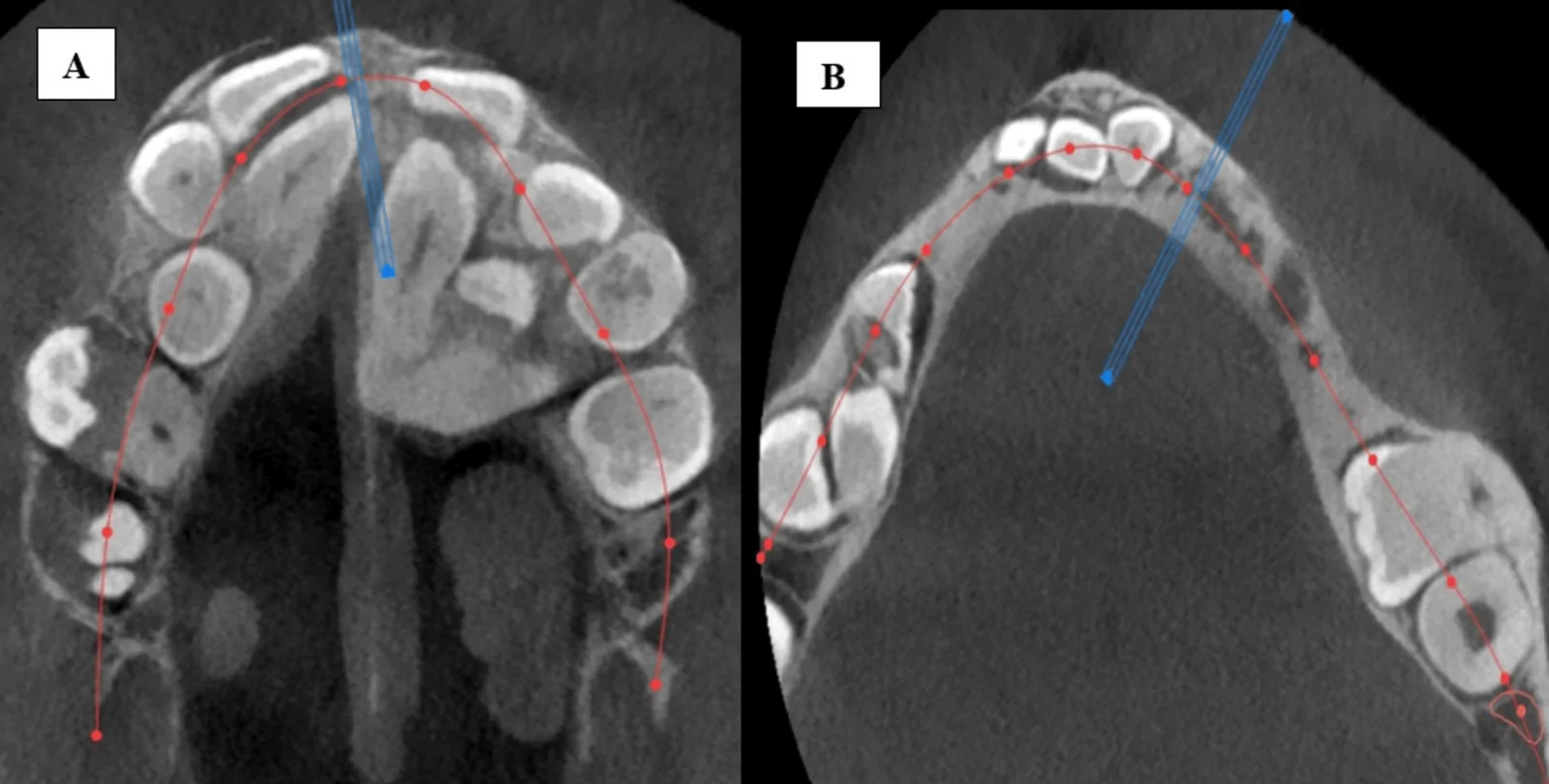
CBCT axial section revealed multiple impacted teeth; A) Maxilla and B) Mandible CBCT: cone-beam computed tomography

CBCT coronal section revealed maxillary sinus hypoplasia, horizontally impacted left maxillary canine, and asymmetry of left inferior turbinate (Figure 3).

**Figure 3:**
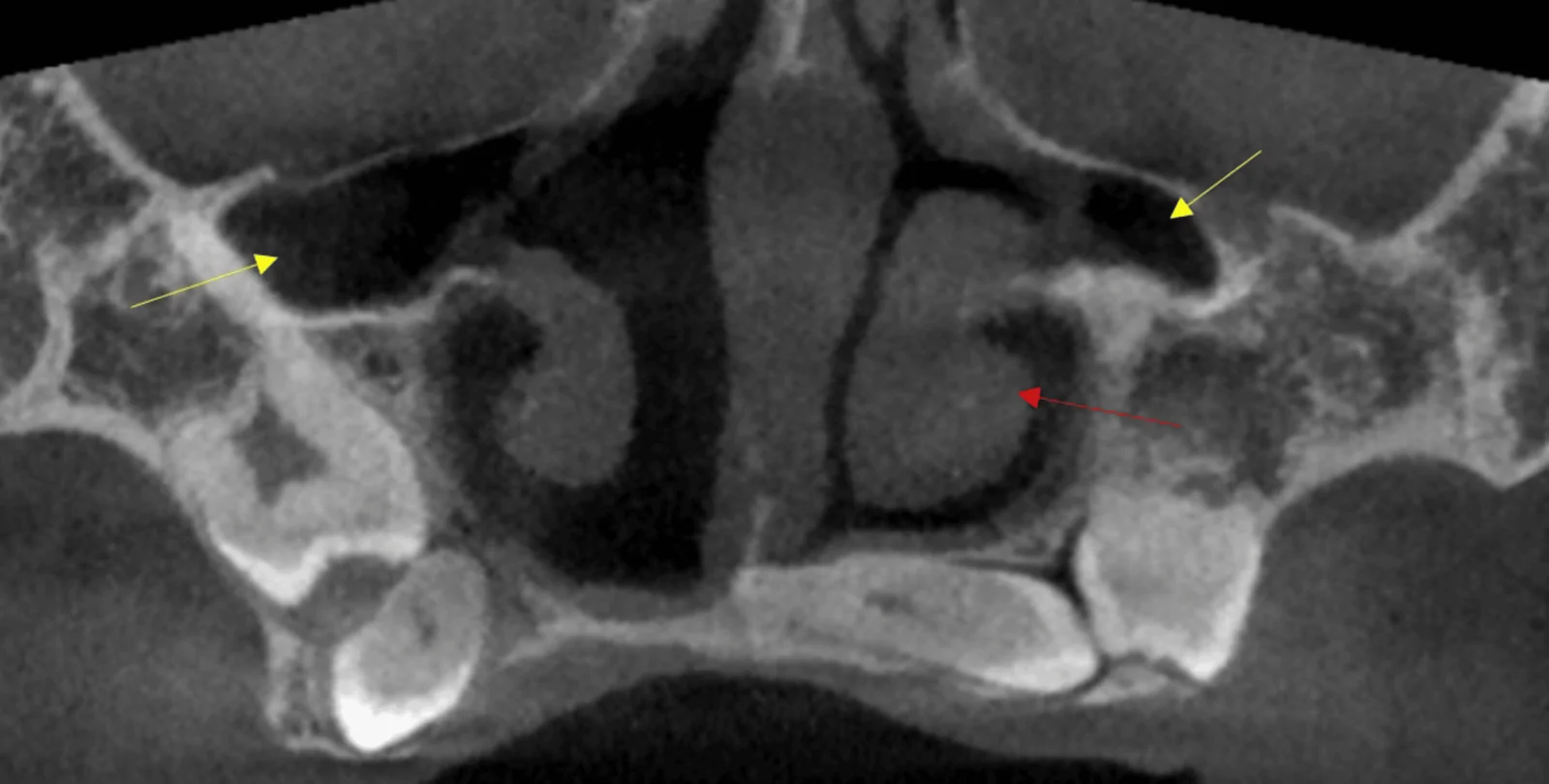
CBCT coronal section revealed maxillary sinus hypoplasia (yellow arrow) and asymmetry of left inferior turbinate (red arrow) CBCT: cone-beam computed tomography

The impacted maxillary second molar was radiologically evaluated three-dimensionally by CBCT in coronal, sagittal, and three-dimensional reconstructed CBCT images, which revealed an acute sharp bend or curvature in the root apex, suggestive of a dilacerated root (Figures 4A-4C).

**Figure 4:**
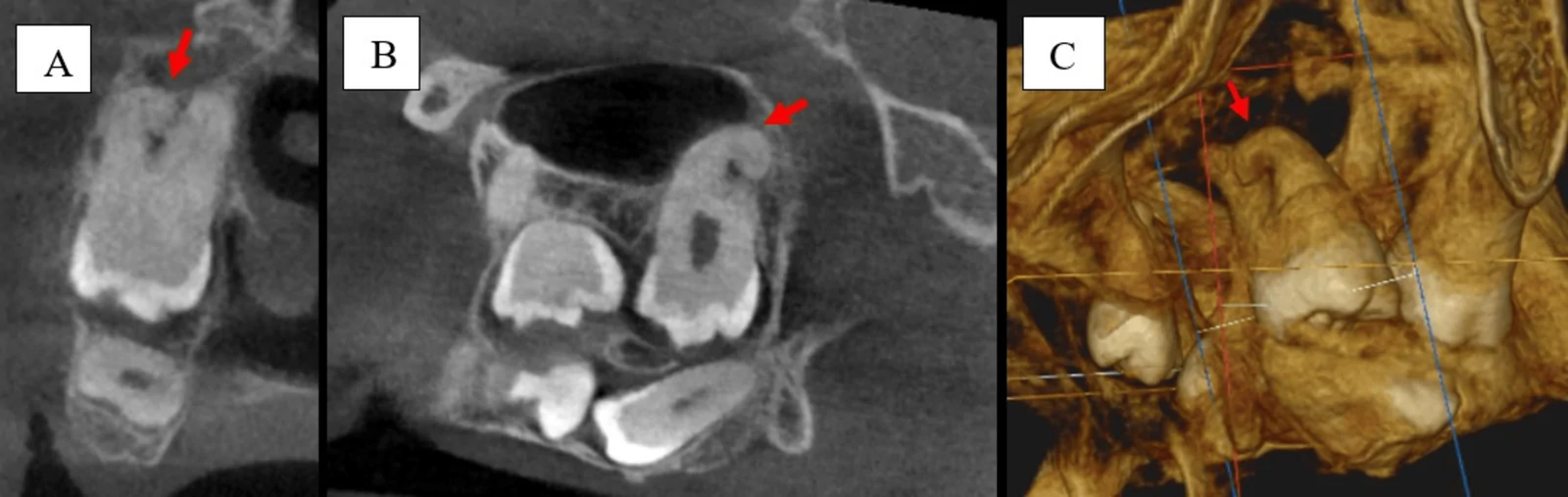
A. CBCT coronal section B. CBCT sagittal section C. 3D reconstructed CBCT image showing dilacerated root (red arrow) CBCT: cone-beam computed tomography

Further radiological evaluation by CBCT revealed regional odontodysplasia or rootless teeth (ghost teeth) in relation to the right mandibular first molar region (Figures 5A, 5B).

**Figure 5:**
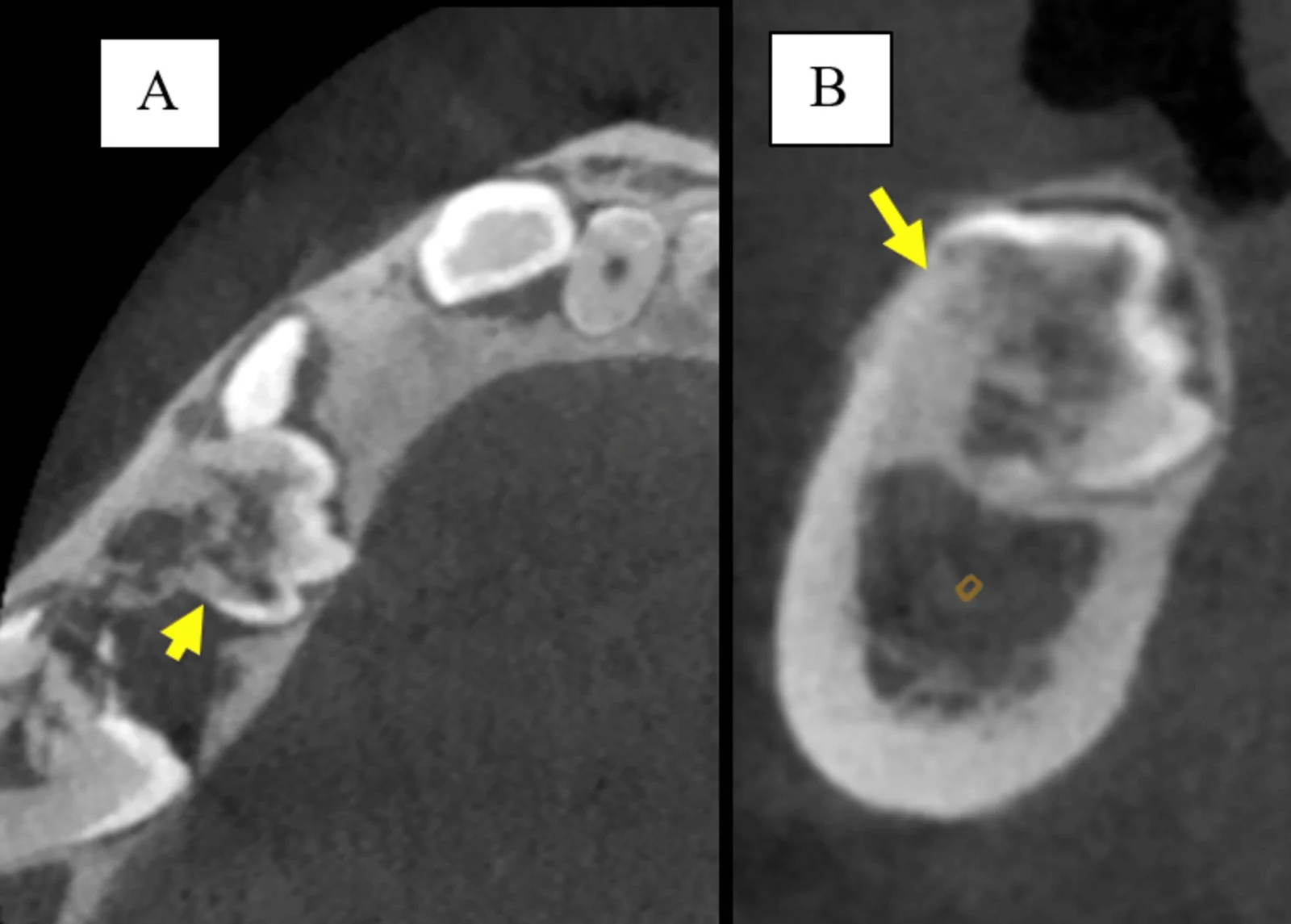
A. CBCT axial section and B. Coronal section revealed ghost teeth (rootless teeth) (yellow arrow) CBCT: cone-beam computed tomography

Three-dimensional reconstructed CBCT images of the maxilla and mandible (Figures 6A, 6B).

**Figure 6:**
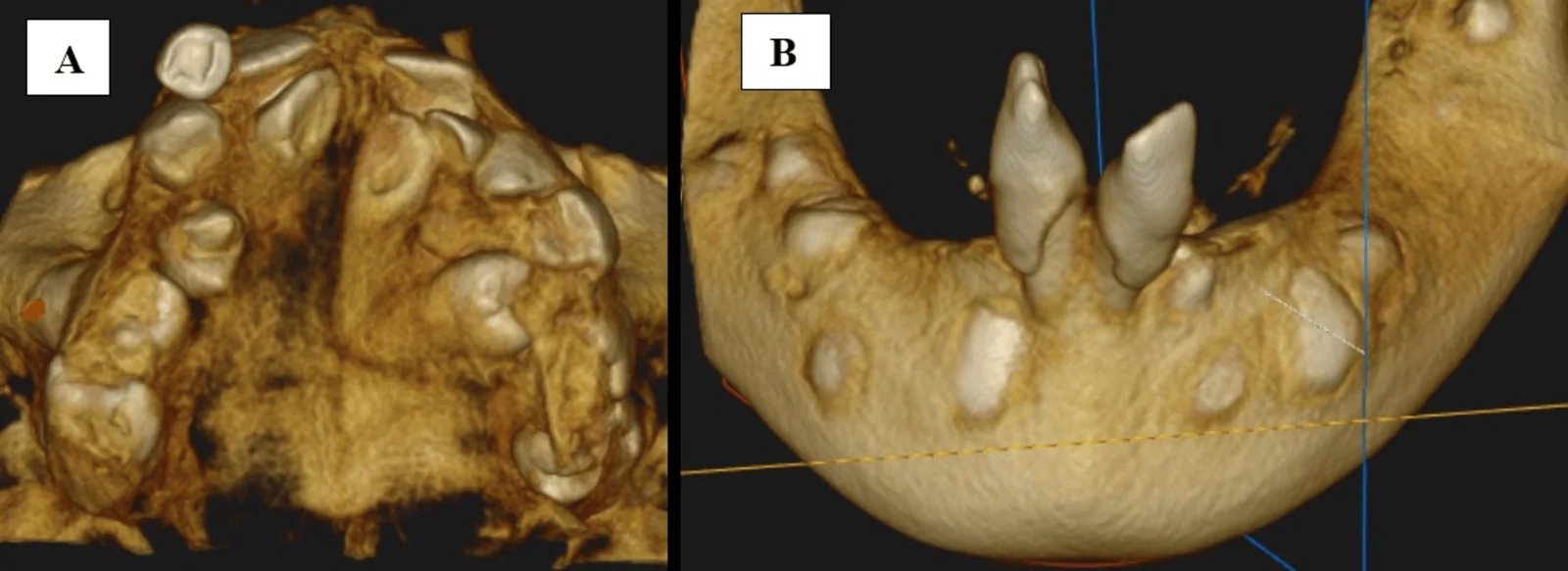
Three-dimensional reconstructed CBCT images showing multiple impacted teeth within the maxilla (A) and mandible (B)

Based on the above radiological findings, a final diagnosis of hyperdontia due to multiple impacted and supernumerary teeth along with dilaceration of impacted right maxillary second molar and regional odontodysplasia (Ghost teeth) of the right mandibular first molar.

Clinical significance

The case report describes an incidental finding of a total of 27 multiple impacted teeth along with dilaceration and regional odontodysplasia (ghost teeth) in a 29-year-old male, which was radiologically evaluated by CBCT.

Limitation

The limitation of this case report is that the intraoral clinical photograph was not provided, as the patient did not give consent for the clinical photograph due to the unesthetic appearance of the oral cavity without teeth. The patient did not turn out for further treatment and follow-up.

## Discussion

Impacted teeth often result from insufficient space in the jaw for proper eruption. Local factors, such as crowded teeth, thick fibrotic gingiva, or abnormal tooth development, can contribute to this condition. Genetically-linked syndromes and diseases associated with multiple impacted teeth include Apert, Down, Cleidocranial dysplasia, Crouzon, Aarskog, Curtius, Zimmerman-Laband, Ehler-Danlos, Noonan's, Fabry-Anderson Fucosidosis, GAPO (Growth retardation, Alopecia, Pseudoanodontia, and Optic atrophy), Hallermann-Streiff, Oro-facial-digital, Osteoglophonic dysplasia, Kippel-Trénaunay-Weber, Nance-Horan, Gardner, Gorlin-Sedano, Yunis-Varon, Singleton-Merten, trichorhinophalangeal (Gleidon), mucopolysaccharidoses, osteopetrosis, and progeria. Endocrine disorders, such as hypothyroidism and hypoparathyroidism, also lead to multiple impacted teeth [1,2]. The literature reviews case reports on multiple impacted teeth are described (Table 1).

**Table 1:** Literature reviews on case reports of multiple impacted teeth

Author	Year	Age/gender	Clinical features
Moturi K, Kalia V [10]	2018	19-year-old female	Multiple dentigerous cysts associated with multiple impacted teeth
Al-Iryani GM, Ali FM [11]	2017	16-year-old female	Multiple impacted supernumerary teeth were reported in patients without syndromes.
Qin B, Jiang W, Jing Z, Zhao L [12]	2016	23-year-old male	Multiple impacted teeth are rare
Sujatha G, Sivapathasundharam B, Sivakumar G, Nalinkumar S, Ramasamy M, Prasad TS [13]	2012	17-year-old male	Multiple missing teeth with compromised aesthetics
Bayar GR, Ortakoglu K, Sencimen M [14]	2008	21-year-old male -2; 20-yr-old male -1	Multiple impacted teeth have varied etiology

Clinical features

The impacted teeth may not cause noticeable symptoms and are only identified through dental imaging. The symptoms of impacted teeth may occasionally include pain, swelling, and tenderness around the affected area [9].

Differential diagnosis

When a dentist observes a lesion with an impacted tooth, the following entities should be evaluated first in the differential diagnosis [15]. Dentigerous cysts, calcifying odontogenic cysts, unicystic (mural) ameloblastomas, ameloblastomas, ameloblastic fibromas, adenomatoid odontogenic tumors, keratocyst odontogenic tumors, calcifying epithelial odontogenic tumors, ameloblastic fibro-odontomas, and complex and compound odontomas [15]. Based on the radiographs of patients, dental practitioners will be able to provide more accurate diagnoses and develop better treatment initiatives [15].

**Table 2:** Lesions associated with impacted teeth Modified table with permission from author [15]

Lesions	Most commonly impacted teeth
Dentigerous cysts	Mandibular third molars (65%), followed by the maxillary canines, maxillary third molars, and mandibular second premolars
Calcifying odontogenic cysts	Canines, maxillary third molar
Unicystic ameloblastomas	Mandibular third molars
Ameloblastomas	Mandibular molars
Ameloblastic fibromas	Premolars and molars
Adenomatoid odontogenic tumors (AOT)	Maxillary canines (40% to 60% of cases), lateral incisors, and mandibular premolars
Keratocystic odontogenic tumours	Mandibular third molars
Calcifying epithelial odontogenic tumor (Pindborg tumor)	Mandibular third molars
Ameloblastic fibro-odontomas	Maxillary and mandibular third molars
Complex odontoma	Mandibular third molar
Compound odontoma	Maxillary incisor

Diagnosis

The dentist can diagnose impacted teeth through clinical examination and radiological imaging techniques by conventional extraoral radiographic views such as intraoral periapical radiograph, occlusal radiograph, anteroposterior skull view, orthopantomography, and specialized imaging techniques like CT and CBCT scans. These imaging modalities help determine the impacted teeth' exact location, position, size, and orientation [9]. Orhan stated that integrating artificial intelligence software tools in cone-beam computed tomography has improved the precision of the impacted teeth' location and the number of root canals present in them [16].

Treatment

The treatment of multiple impacted teeth depends on various components, including the position and type of the impacted teeth, the patient's age, and the presence of clinical signs and symptoms. Treatments include monitoring in some cases; impacted teeth may be monitored over time, especially if they are not causing any issues. Extraction is indicated for impacted teeth, particularly wisdom teeth. Surgical extraction may be necessary for impacted teeth deeply embedded in the jawbone. Orthodontic Intervention employs mechanics based on cantilever springs, the micro-implant anchorage, to create forces to erupt impacted teeth [16]. Such interventional treatment is recommended to create more space in the jaw and guide the eruption of impacted teeth. Piezosurgery combined with highspeed contra-angle handpiece aids in better removal of obliquely placed impacted supernumerary teeth [17]. For the impacted teeth caused by the maxillary insufficiency in skeletal class III malocclusion, digital orthodontic appliances designed and manufactured with CADCAM technology, such as three-dimensional printed laser-sintered orthodontic appliances for rapid maxillary expansion are used [18]. Brinkman et al. suggested placing fixed prostheses by implant-supported abutments driven through the root of impacted teeth without extraction [19].

Complications

If left untreated, impacted teeth can lead to various complications such as pericoronitis infection, cyst formation, damage to adjacent teeth, and pathological migration or changes in the alignment of other teeth.

## Conclusions

Multiple impacted teeth are usually identified as an incidental finding by radiological evaluation by CBCT. It is crucial to consult with a dentist or oral surgeon for multiple impacted teeth. A multidisciplinary approach should be used to manage the case of multiple impacted teeth, which requires an oral surgeon, an orthodontist, a prosthodontist, and a pediatric dentist to be involved. In these cases, serial examinations are conducted on the primary teeth, supernumerary teeth are removed, and surgical exposure and orthodontic intervention by springs force the eruption of impacted teeth that cannot erupt. Such interventions provide a comprehensive evaluation and recommend an appropriate treatment plan based on the situation. Early detection and intervention can help prevent potential complications associated with impacted teeth.
